# A national survey of oral maxillofacial surgeons' and trainees' awareness and practice regarding psychological problems associated with facial trauma

**DOI:** 10.4314/ahs.v22i4.22

**Published:** 2022-12

**Authors:** Olufemi Kolawole Ogundipe, Ayodele Gbenga Akomolafe, Adewale Francis Adejobi, Azuka Raphael Njokanma, Adesanmi Akinsulore

**Affiliations:** 1 Department of Oral and Maxillofacial Surgery, Faculty of Dentistry, Obafemi Awolowo University Ile-Ife; 2 Department of Oral and Maxillofacial Surgery, Obafemi Awolowo University Teaching Hospital Complex (OAUTHC), Ile-Ife; 3 Department of Mental Health, Faculty of Clinical Sciences, Obafemi Awolowo University Ile-Ife

**Keywords:** Maxillofacial trauma, psychological problems, Nigeria

## Abstract

**Background:**

The psychological problems associated with facial trauma may sometimes pose additional health concerns for the injured patient. Understanding the gaps in the Oral and Maxillofacial surgeons' (OMFS) awareness of patient in need of specialist mental health intervention is important in ensuring holistic care for the trauma patient.

**Objectives:**

To determine the knowledge, practices and self-assessed level of competence of Nigerian Oral maxillofacial surgeons/trainees regarding psychological problems associated with facial trauma and to determine their perceived need for training in assessment of psychological consequences following facial trauma.

**Methods:**

A cross-sectional study was conducted in which a web-based structured questionnaire was sent to Oral and maxillofacial surgeons and trainees.

**Results:**

Majority of respondents (85.2%) had encountered psychological problems in patients who have sustained facial trauma. Half (50.6%) of OMFS / trainees had high level of knowledge regarding psychological problems associated with facial trauma; depression, anxiety, post-traumatic stress disorder, body image disorder and acute stress syndrome were the five most common trauma related psychological problems mentioned. More respondents reported low level of competence in conducting mental state examination when compared to other skill sets. In-unit counselling was reported as the method of intervention by majority (69.1 %), followed by referral to the mental health specialists (17.3%). More than one third (40.7%) of OMFS were extremely interested in undergoing additional training in the psychological assessment of patients who have sustained facial trauma.

**Conclusion:**

Nigerian Oral and maxillofacial surgeons frequently encounter psychological problems in patients with traumatic facial injuries. Notwithstanding their perceived self-assessed low level of competence in psychological assessment of trauma patients, objective assessment revealed a relatively high level of knowledge of psychological problems that can affect the facial trauma patient with many indicating a high perceived need for additional training in the psychological assessment of facial trauma patient. There is a need for incorporating psychological assessment into the management to ensure holistic care of trauma patients.

## Introduction

Patients presenting with facial trauma / injuries are known to display substantial psychological, emotional and behavioural problems in the immediate period following injury and up to one year follow-up.^1^,^2^,^3^ These trauma-related psychological problems have been described as a major public health issue in both low and high income countries.^4^ These are often reactions to stress associated with major facial injuries with accompanying post-injury facial disfigurement although psychological problems have also been reported in patients with minor facial injuries.^5^ Psychological problems following trauma include depression, anxiety, post-traumatic stress disorder (PTSD), alcohol abuse, etc. The intensity of the psychiatric response depends on trauma characteristic and individual vulnerabilities.

Globally, the prevalence of posttraumatic psychological problems ranges from 10–70%.^6^,^7^,^8^, Studies carried out in Nigeria found that patients with facial injuries are at risk of poor quality of life (QoL) after facial trauma with high incidence of depression and anxiety in the follow up period.^9^–^12^

Even though Maxillofacial surgeons are at the forefront of specialist care for patients with traumatic facial injuries, priority is often placed primarily on the physical injuries at the expense of the psychological need of the patient.^13^ This unmet need in diagnosis and treatment of these problems can adversely affect the patients' quality of life after maxillofacial trauma and implies a breach of the principle of beneficence and non-maleficence.^9^,^12^

Among the barriers to psychological care following trauma are non- integration of mental health unit into the maxillofacial trauma team,^14^ low utilization of psychological screening tools, need for care provider training, underestimation of patient's psychological need and lack of relevant knowledge of posttraumatic psychological sequelae by the surgeon in developing countries.^14^,^15^

A multinational survey of OMFS in France, UK and US revealed adequate relevant knowledge in posttraumatic psychological care but varied referral patterns and practice protocols.^7^

Thus far, the knowledge and practices regarding post traumatic psychological assessment of Nigerian Oral and maxillofacial surgeons has not been studied. The frequency with which Nigerian OMFS encounter psychological problems in patients following facial trauma has rarely been examined and there is a paucity of nationally representative data on the knowledge and practice of OMFS regarding psychological problems following facial trauma. Consequently, the primary aim of this survey was to determine the frequency with which Nigerian OMFS encounter psychological problems in facial trauma patients and their knowledge of post-facial trauma psychological problems.

A secondary purpose of this survey was to assess their perceptions of their competence in assessing post-traumatic psychological problems and need for further training.

## Methods

This study was designed as a cross-sectional study and was conducted between March and May 2021 among OMFS and trainees spread across the six geopolitical zones (North-east, North-west, North-central, Southwest, South-east and South-south) of Nigeria.

A census sampling method was used in the selection of participants in this study. A link to the web-based anonymous questionnaire was sent to the official WhatsApp group of the Nigerian Association of Oral Maxillofacial surgeons (NAOMS) with a covering letter describing the purpose of the survey. The WhatsApp group is very popular and well subscribed among members of NAOMS because it is the major platform used for information dissemination. The modified questionnaire by Pitak-Arnnop et al.^7^ was used in this study and consisted of a mixture of open-ended, dichotomous, objective structured and Likert responses. Part A of the questionnaire assessed socio-demographic status, while part B assessed four domains; awareness & knowledge, competence in assessment and management, practice and interest in additional training. Follow-up reminders were sent to the NAOMS WhatsApp group at the end of week 1, 2, 3, 4 and 6 after initial contact. In this study, inclusion criteria were all OMFS and trainees who were registered in the WhatsApp group of NAOMS while those unwilling to give consent were excluded from the study. Respondents were required to include their e-mail address in the questionnaire to track multiple responses. The recommendations of the ‘Helsinki declaration’ was maintained during this study as participants were required to give informed consent and the anonymity of all participants was respected.

### Study variables

The outcome variables included; (1) the frequency of encountering post traumatic psychological problems, (2) knowledge of symptoms of post-traumatic psychological problems, (3) self -assessed competence of surgeons in assessment and management of posttraumatic psychological problems, (4) practice of surgeons in assessment and management of post-traumatic psychological problems, and (5) perceived need for additional training in diagnosis and management. The awareness and knowledge domain consisted of two open-ended questions, one question requiring a dichotomous response and another requiring objective structured responses. The knowledge domain consisted of 10 multiple choice questions. A score of 0 to 10 was computed as; Low (0–3), Moderate (4–6), and High (7–10). The self-assessed competence, practice and additional training need domains consisted of 5 questions requiring Likert-like responses.

The questionnaire was pretested with an independent group of four OMFS to calibrate the questionnaire and assess how it addressed the questions on the five outcome variables. Subsequently, adjustments to the questionnaire were made and it was tested on another group of four OMFS.

### Data analysis

The data from the returned questionnaires were entered into a database, crosschecked, and then analysed with SPSS (Version 21, New York, USA) using simple descriptive statistics.

## Results

### Demographics

One hundred and fifty-two OMFS and trainees practicing in various hospitals across the 6 geo-political zones in Nigeria were surveyed. Of these, 53.3% (n=81) responded. Overall, 82.7% of the respondents were males while 17.3% were female. Eighty- six percent (86.5%) of the respondents have their practice in teaching hospitals, 4.9% in federal medical centers and 8.6% in a private Hospitals. The South Western part of Nigeria had the highest concentration of respondents (67.9%) followed by North West (14.8%), North Central (11.1%), South-South (3.7%) and North East (2.5%). (Table 1).

**Table 1:** Socio-demographic characteristics and results of objective assessment of the level of knowledge of common psychological problems associated with facial trauma among Oral and Maxillofacial surgeons in Nigeria

Variables	Frequency (N=81)	%
**Gender**		
Male	67	82.7
Female	14	17.3
**Place of Practice**		
Teaching Hospital	70	86.7
Federal Medical Center	04	4.9
Private Practice	07	8.6
**Zones**		
North west	12	14.8
North East	02	2.5
North Central	09	11.1
South West	55	67.9
South South	03	3.7
South East	0	0.0
**Total**	**81**	**100**
**Level of** **knowledge**	**Frequency (N=81)**	%
Low (0–3)	2	2.5
Moderate (4–6)	38	46.9
High (7–10)	41	50.6
**Total**	**81**	**100**

**Figure 1:**
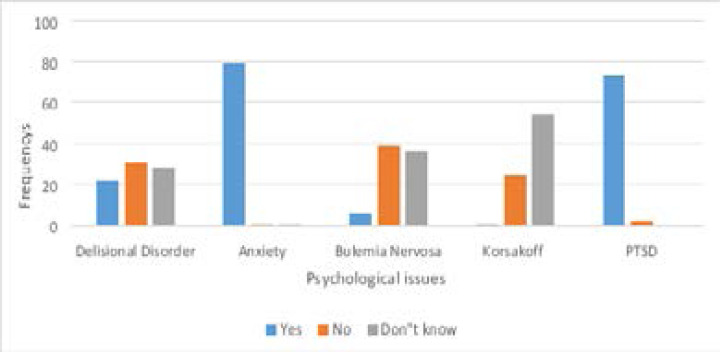
Objective assessment of OMFS knowledge of psychological problems that affect the facial trauma patient

**Figure 2:**
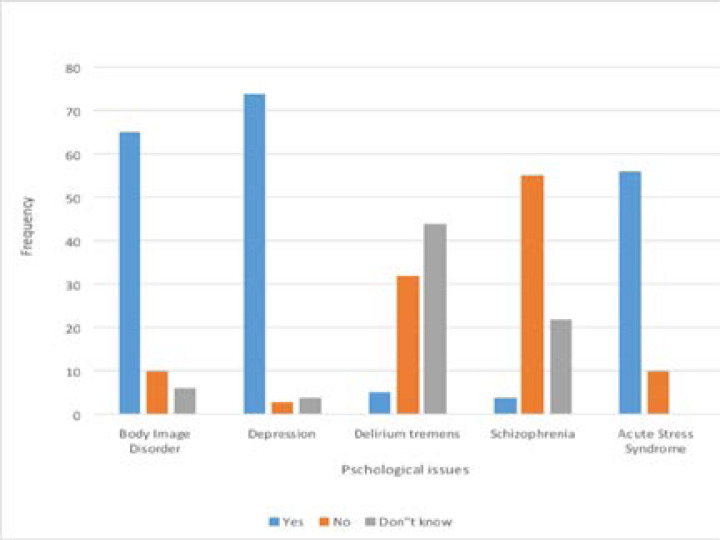
Objective assessment of OMFS knowledge of psychological problems that affect the facial trauma patient.

The frequency of encountering psychological problems was high among OMFS. Sixty-nine respondents (85.2%) reportedly encountered psychological problems in patients with facial injuries, while only 12 respondents (14.8%) reported otherwise. Objective assessment of the awareness of OMFS showed moderate-high (97.5%) awareness of possible psychological problems that may be associated with facial trauma (Table 1). Depression, anxiety, post-traumatic stress problems, denial and body image problems due to aesthetic and functional challenges were some of the psychological problems identified by the respondents. (Figure 1 and 2)

In the competence domain, 23 (28.4 %), 53 (65.4%), 16 (19.8%), 45 (55.6%), 30 (37.1%) of the respondents rated themselves as having low level of competence in eliciting symptoms of psychological problems, conducting mental state examination, conducting neurological examination, making a psychological diagnosis, and providing psychological care such as counselling and diagnosing while 14 (17.3%), 5 (6.2%), 20 (24.6%), 5 (6.1%), 7 (8.6%) rated themselves as having high level of competence with the remaining rating themselves as having moderate level of competence. Similarly, twenty (24.7%) and twelve (14.8%) of the respondents reported that they routinely conduct neurological examination and offer psychological counselling to facial trauma patients in their practice. Therefore, their perceived self-assessed level of competence and practice were generally low for all the five skill sets examined. (Table 2).

**Table 2:** Self-assessed competence and practice of OMFS regarding diagnosis and management of psychological problems following facial trauma.

	Competence Domain	Low Freq (%)	Moderate Freq (%)	High Freq (%)	Total (Freq)
**1**	Eliciting symptoms of psychological problems	**23 (28.4)**	**44 (54.3)**	**14 (17.3)**	**81**
**2**	Conducting mental state examination	**53 (65.4)**	**23 (28.4)**	**5 (6.2)**	**81**
**3**	Conduction neurological examination	**16 (19.8)**	**45 (55.6)**	**20 (24.6)**	**81**
**4**	Making a psychological diagnosis	**45 (55.6)**	**31 (38.3)**	**5 (6.1)**	**81**
**5**	Providing psychological care such as counselling	**30 (37.1)**	**44 (54.3)**	**7 (8.6)**	**81**

	Practice and attitude Domain	Never Freq (%)	Rarely Freq (%)	Sometimes Freq (%)	Often Freq (%)	Total
**1**	Eliciting symptoms of psychological problems	**12 (14.8)**	**20(24.7)**	**44 (54.3)**	**5 (6.2)**	**81**
**2**	conducting mental state examination	**20 (24.7)**	**32 (39.5)**	**26 (32.1)**	**3 (3.7)**	**81**
**3**	Conduction neurological examination	**9 (11.1)**	**13 (16.1)**	**39 (48.1)**	**20 (24.7)**	**81**
**4**	Making a psychological diagnosis	**16 (19.8)**	**30 (37.0)**	**31 (38.3)**	**4 (4.9)**	**81**
**5**	providing psychological care such as counselling	**8 (9.9)**	**14 (17.3)**	**47 (58.0)**	**12 (14.8)**	**81**

In this study, the method of intervention frequently employed was in-unit counselling by 56 (69.1%) of the respondents. Only 14 (17.3%) of the respondents referred their patients for Psychiatrist evaluation.

Respondents were asked about their perceived need for further training and eighty four percent of the respondents reported that they have not had any formal training in identifying psychological problems associated with facial trauma. Respondents were also asked for their level of interest in acquiring additional training in diagnosing psychological problems, majority of the respondents were extremely/very interested (Table 3).

**Table 3:** Oral and Maxillofacial Surgeon's interest in additional psychological training

	Interest in additional training Domain	extremely interested Freq (%)	very interested Freq (%)	moderately interested Freq (%)	slightly interested Freq (%)	not interested Freq (%)	Total Freq (%)
**1**	Eliciting symptoms of psychological problems	**29 (35.8)**	**37 (45.7)**	**12 (14.8)**	**0 (0.0)**	**3 (3.7)**	**81**
**2**	conducting mental state examination	**31 (38.8)**	**32 (39.5)**	**13 (16.1)**	**2 (2.5)**	**3 (3.7)**	**81**
**3**	Conduction neurological examination	**39 (48.1)**	**30 (37.0)**	**8 (9,9)**	**0 (0.0)**	**4 (4.9)**	**81**
**4**	Making a psychological diagnosis	**33 (40.7)**	**34 (42.0)**	**10 (12.4)**	**0 (0.0)**	**4 (4.9)**	**81**
**5**	providing psychological care such as counselling	**40 (49.4)**	**31 (38.3)**	**5 (6.2)**	**1 (1.2)**	**4 (4.9)**	**81**

## Discussion

The study aimed to determine the knowledge and practice of Nigerian OMFS regarding post-traumatic psychological problems using a survey method. To the best of our knowledge, this is the first Nigerian study that has surveyed OMFS in all regions of the country on their awareness and practice regarding psychological problems in facial trauma patients.

The response rate was average at 53.3%. This is much higher than the average response rate for online surveys (32.6 to 33.0%) recorded in most studies^16^,^17^. In addition, overwhelming majority of the respondents were male practicing in teaching hospital of the South West of Nigeria which precluded use of inferential statistics. The findings of the present study should be interpreted with this in mind.

The practice of OMFS in Nigeria is male dominated. This may be due to cultural and societal demands of females in Nigeria. Xepoleas et al.^18^ agreed to our presumption judging from their conclusion in a review article titled ‘The experience of female surgeon around the world, scoping review’. Overwhelming majority of OMFS were from South western, Nigeria. This finding is as a result of large clusters of maxillofacial centers in this region compared to the other five geopolitical zones.

In this study, 85.2 % of OMFS reported that they have encountered facial trauma patients with psychological problems which suggests a high self-reported prevalence of this condition. Other studies have reported prevalence of 10 to 48% of this condition 19–21. This includes a Nigerian study, where they reported depression in 48% of patients at baseline and in 22% of patients at 10–12 weeks after facial trauma^21^. The present survey of the opinions of OMFS is in agreement with the above studies. The variability in prevalence among societies has been attributed to the availability of post trauma social support, previous mental health illness, presence of post-operative pain or discomfort and substance abuse^22^. Interestingly, 14.8% of surgeons in this study reported not to have encountered a facial trauma patient with psychological disorder. This may be as a result of absence of psychological ailment in these patients but calls to question if the diagnosis was missed in patients who actually needed psychological care. The maxillofacial surgeon as a primary caregiver plays the role of a gatekeepers to the patients who have sustained traumatic facial injuries. It is the duty of the primary caregiver to identify when there is a need for referral, as prompt and appropriate referral may affect the process of patient evaluation, treatment outcomes and cost. When objectively assessed, depression, anxiety and post-traumatic stress problems were mostly identified by the respondents in our study. Similarly, Bisson et al.^23^ in their study reported that patients had a 27% likelihood of progressing to develop post-traumatic stress disorder approximately 7 weeks after the facial injury occurred. Shepherd et al.^24^ also reported the development of depression, anxiety problems and psychological stress in patients within 3 months of sustaining a mandibular fracture. Levine et al.^25^ in their study, reported significantly higher incidence of post-trauma unemployment, marital problems, binge drinking, jail, and lower attractiveness scores in patients with traumatic facial disfigurement. These were not assessed in this study.

Most respondents (69.1 %) in this study employed in-unit counselling as a method of management while only 17.3% of respondents regularly referred their patients to the mental health physician for management. Various studies^26^,^27^ have reported a low rate of psychiatric referral with psychiatric problems often under-recognized, under-diagnosed and therefore poorly treated. The reasons for the low referral rate in this study may be linked to non-recognition of the psychiatric morbidity of patients, as most respondents (55.6%) reported a low level of knowledge in diagnosing psychological ailments. Other reasons for low referral reported in the literature include: uncertainty of the beneficial effect of such referral, concerns about the effect of a psychiatric referral on the self-esteem of patients, and scarcity of psychiatric specialist care^28^,^29^.

In Nigeria, Oral and Maxillofacial surgery is a dental degree-based specialty. This has some similarities to what obtains in the United State of America however, a qualification in Medicine may be undertaken optionally during residency training in the USA. In contrast, in the United Kingdom, Oral and Maxillofacial surgery is a medical based specialty. This also implies variation in curriculum and degree of exposure to training in mental health based on the country where the surgeon obtains his training.

Most maxillofacial surgeons reported interest in acquiring additional level of training in diagnosing psychological problems with 33 (40.7%) respondents extremely interested, with only 4(4.9%) surgeons indicating no interest. This shows a high interest amongst maxillofacial surgeons in acquiring additional training necessary to recognize, diagnose and appropriately refer patients who may need expert management of their mental health.

## Conclusion

Nigerian Oral maxillofacial surgeons frequently encounter psychological problems in patients with traumatic facial injuries. OMFS in Nigeria demonstrated a relatively high level of knowledge of psychological problems that may affect the facial trauma patient. Conversely, their self-assessed level of competence was low. There is a high perceived need for additional training in diagnosis and management of these patients among OMFS in Nigeria. Incorporation of mental health in the training curriculum of OMFS will be beneficial to aid the diagnosis and appropriate referral of facial trauma patients in need of psychological care.

## Figures and Tables

**Figure 3:**
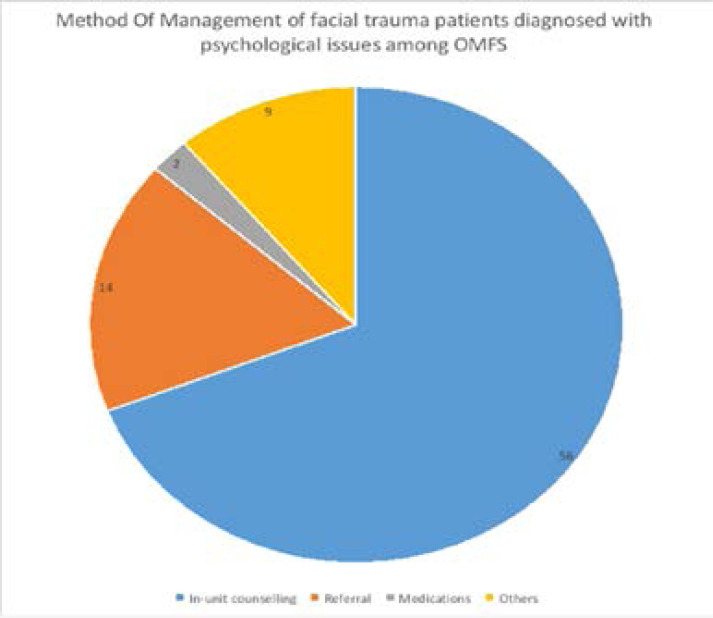
Method of management of facial trauma patients diagnosed with psychological problems among OMFS in Nigeria
